# Analysis of the Effectiveness of Arytenoidectomy and Posterior Cordectomy with the Use of CFD Airflow Measurements in Patients with BVFP: A Retrospective Study

**DOI:** 10.1155/2022/9749034

**Published:** 2022-11-15

**Authors:** Marta Gamrot-Wrzoł, Magdalena Marków, Daniel Janecki, Bogusława Orecka, Krzysztof Warmuziński, Maciej Misiołek

**Affiliations:** ^1^Department of Otorhinolaryngology and Laryngological Oncology in Zabrze, Medical University of Silesia, Zabrze, Poland; ^2^Department of Process Engineering, University of Opole, Opole, Poland; ^3^Institute of Chemical Engineering, Polish Academy of Sciences, Gliwice, Poland

## Abstract

**Purpose:**

Bilateral vocal fold paralysis (BVFP) is a rare larynx disease manifested by dyspnea, which often requires surgical treatment. The aim of the study is to determine the effectiveness of unilateral arytenoidectomy with posterior cordectomy in the treatment of BVFP using the computational fluid dynamics (CFD) method.

**Methods:**

This study included 33 patients with BVFP who underwent unilateral laser arytenoidectomy with posterior cordectomy. Glottis area measurements and spirometry, as well as a self-assessment of respiratory efficiency were performed before the surgery and after the recovery period. Using the CFD method, computer models of the glottis were made. Then, changes in air pressure gradient and maximum air velocity at the level of glottis were calculated, and local fields of pressure and air velocities were obtained.

**Results:**

The values of glottal surface area (S), spirometry parameters (forced expiratory volume in one second (FEV_1_), forced vital capacity (FVC), and peak expiratory flow (PEF)), inlet air velocity at the glottal level as well as patients self-assessment of respiratory efficiency turned out to be significantly higher after the operation. The values of maximum velocity at the glottal level, pressure gradient at the glottal level turned out to be significantly lower after the surgery. We observed that the greater the increase in glottal surface area, the greater the decrease in self-assessment scales (visual analogue scale (VAS) and Medical Research Council (MRC)). Increased levels of spirometry parameters after the surgery correlated with smaller decrease of PEF-dependent pressure gradient at the glottal level (PEF*ΔP*_CFD_).

**Conclusion:**

Unilateral laser arytenoidectomy with posterior cordectomy is an effective method for the treatment of BVFP. CFD is a useful tool to determine and visualize the effectiveness of surgical treatment in BVFP.

## 1. Introduction

Bilateral vocal fold paralysis (BVFP) is a laryngeal condition that involves immobilization of the vocal folds in paramedian or, less commonly, intermediate position due to recurrent laryngeal nerve (RLN) damage. It is usually manifested by dyspnea, dysphagia, and dysphonia of varying severity. Exercise-induced dyspnea usually occurs when the glottal area reduces by at least three-fourths. Adaptation to reduced respiratory surface area is individual and depends on many factors (obesity, cardiovascular, and respiratory diseases) [1–3]. So far, the assessment of respiratory conditions in BVFP has been possible using spirometry.

Computational fluid dynamics (CFD) is a specialistic branch of physics devoted to fluid balance and motion, as well as their effects on both boundaries and immersed bodies (according to the Navier−Stokes equation). Here, the term fluid includes both liquids and gases, since these bodies share a common feature—inability to maintain shape. When deriving the equations for fluid motion, shear stress on the walls of viscous fluid element is also considered in addition to normal stress. As a result, CFD enables approximate estimation of the distribution of velocity, pressure, temperature, and other parameters in a flowing fluid. This is why the CFD method, more precisely than spirometry, can show the characteristic of human body airflow. Upper airway models, needed for CFD simulation, are created based on computed tomography (CT) or magnetic resonance imaging (MRI) scans and videolaryngoscopy pictures. Their use allows for the imaging of the mechanisms and effects of various pathologies, as well as the outcomes of potential therapeutic interventions within the upper respiratory tract (URT) [4, 5]. This feature made the CFD method unique compared to other commonly used diagnostic techniques—it enabled researchers to observe changes in airflow parameters in conditions leading to glottic stenosis, such as vocal cord dysfunction (VCD), i.e., paradoxical vocal fold movement [6], subglottic stenosis [7], congenital glottic web [4], or BVFP [5, 8, 9].

So far, no comprehensive analysis of CFD in the context of pre- and postoperative outcomes in patients with BVFP has been reported, which encouraged our own research.

## 2. Materials and Methods

**Figure 1:**
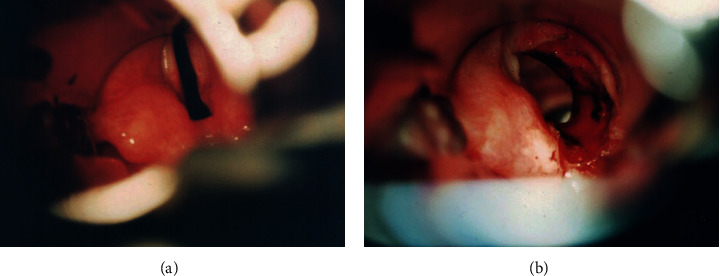
Intraoperative endoscopic view before (a) and after (b) the right arytenoid cartilage and posterior one-third of the vocal cord removal.

Patients with BVFP receiving surgical treatment in the Department of Otorhinolaryngology between 2014 and 2018 were included in the analysis. Unilateral laser arytenoidectomy with posterior cordectomy was performed under general anesthesia with the Kleinsasser set for laryngeal microsurgery, using a Zeiss OPMI Movena microscope and a Dream Pulse CO_2_ laser (minimal spot size at a focal length of 400 mm; continuous-wave mode with power setting of 10 W). To minimize laser-specific hazards during CO_2_-laser microsurgery, the armored endotracheal tubes were used. During the procedure, the arytenoid cartilage and posterior one-third of the vocal cord were removed unilaterally (Figure 1). This relieved the tension of the glottic sphincter, retracted the thyroarytenoid muscle and the vocal fold anteriorly, and led to the widening of the posterior airways.

Mitomycin C (MMC) was used intraoperatively—an MMC solution (1 vial, 10 mg) was prepared at the end of the surgery in 5 mL of saline and was applied on the postoperative wound for about 2 min using a swab holder. All patients postoperatively received intravenous steroids (dexamethasone) followed by oral steroids after discharge from the department, with gradual dose reduction until discontinuation of the drug. Antibiotics and antireflux agents were not administered.

### 2.1. Study Group

A total of 33 patients (30 females and 3 males) were included in the analysis. RLN injury occurring as a complication of strumectomy was the most common cause of BVFP (29 patients—4 operated on for papillary carcinoma, others for nodular goiter). In three cases, BVFP was caused by prolonged intubation. The cause of paralysis remained unknown (idiopathic) in one case.

Patients were qualified for surgery approximately 1 year after the onset of BVFP. Laser arytenoidectomy with posterior cordectomy was the first and the only glottis-dilating surgery in most patients. Three patients had previously undergone laterofixation, and one patient—partial cordectomy. In the course of the disease, tracheostomy was required in nine patients.

### 2.2. Research Methods

**Figure 2:**
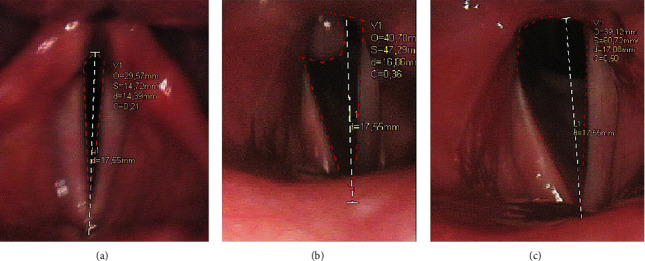
A videolaryngoscopic view of the glottis in Patient 8: (a) before glottis-expanding surgery; (b) 3 months after glottis-expanding surgery—granulation tissue is present in the postoperative scar; (c) 6 months after the procedure—resolution of granulation tissue after conservative management.

Video laryngoscopy was performed with STORZ videolaryngoscope. The image was recorded using the IRIS software and stored in the computer hard disk memory. Photographs of patients' larynx were analyzed at maximum opening of the vocal folds. The contours of the glottis were outlined and the glottis length was standardized by introducing a constant value of 17.55 (±0.92) for women and 20.09 (±3.07) for men, which, according to Eckel and Sittel, are highly repeatable [10]. Then the surface area (S), the circumference of the outlined figure (O), circularity (C), and the length of the segment connecting the two most distal points on the perimeter (d) were calculated automatically by the IRIS program (Figure 2).

Spirometry was performed twice, before and after the procedure, in each patient. The following parameters were assessed: FVC—forced vital capacity, FEV_1_—forced expiratory volume in one second, and PEF—peak expiratory flow. To perform tests in patients after tracheotomy, the tracheotomy tubes were removed and tracheostomy was occluded by external application of a plastic membrane. Special care was taken to prevent any air leakage during examination [15]. In all patients, spirometric measurements were performed on the day of the endoscopic examination. First, before arytenoidectomy and next, following complete healing of the larynx (within 1.5–6 months after the surgical procedure). In case of granulation in the postoperative scar or reoperation needed, included in the study were tests performed after the final healing effect was achieved.

**Figure 3:**
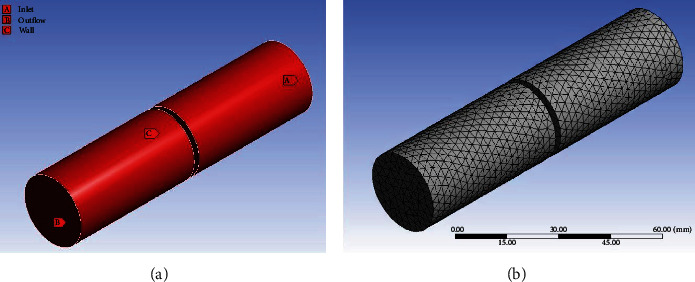
(a) A geometric model of the glottis with boundary conditions; (b) a geometric mesh placed on the geometric model.

Following the example of Marków et al., the results of noninvasive tests, such as videolaryngoscopy and spirometry, were used to create a computer model of each patient's glottis [9]. We developed a geometric model of the glottis along with the adjacent URT segment in collaboration with the Institute of Chemical Engineering of the Polish Academy of Sciences in Gliwice, and using the ANSYS Workbench software. Certain simplifications were adopted to determine the geometric model (Figure 3). It was assumed that the glottal thickness is 2 mm, and that the supra- and subglottic larynx is 50 mm long each. The boundary conditions were as follows: no slip of the fluid at the wall, flat velocity profile at the inlet, and no velocity gradient across the outlet. Since all results were obtained during the expiratory phase, the inlet values are those on the tracheal side.

Numerical simulation of the airflow in the geometric glottic model was performed for each patient using the ANSYS FLUENT package. Glottal area and ventilation parameter measurements were used to calculate airflow velocity, i.e., inlet velocity – *w*_inlet_ (m/s), maximum velocity – *w*_max_ (m/s), and pressure gradient − *ΔP*_CFD_ (Pa) at the glottal level.

### 2.3. Questionnaire

The patients completed a questionnaire on the course of disease, a subjective assessment of the severity of dyspnea 5-point Medical Research Council (MRC) scale and a subjective assessment of breathing comfort (10-point visual analogue scale (VAS)) twice—before and after the procedure, on the same day when other examinations were held (spirometry and endoscopy).

### 2.4. Opinion of the Bioethics Committee

Our study has a retrospective character and was granted permission by the Bioethics Committee of the Medical University of Silesia, No. KNW/022/KB1/227/I/16.

### 2.5. Statistical Analysis

Parametric and nonparametric tests were performed for dependent samples to assess the impact of the surgery on changes in the measured parameters. First, the distribution of the difference in pre- and postoperative parameter values was evaluated. Parametric Student's *t*-tests were used for parameters with difference distribution consistent with normal distribution (assessed based on *W*-statistics significance in the Shapiro−Wilk test) or if the absolute value of skewness was not greater than one |*Sk.*| < 1. Nonparametric Wilcoxon tests based on the *Z* statistics were used for parameters with difference distribution other than normal and characterized by high skewness |*Sk.*| > 1.

Scatter plots for pairs of variables were developed to assess the nature of the relationship between the parameters. Statistical analyses were considered significant if the probability of observing the test statistics for the null hypothesis was lower than or equal to 5% (*p* ≤ 0.05).

## 3. Results

### 3.1. Glottal Area Measurements and Spirometric Parameters

Postoperative increase in glottal area (*S*, mm^2^) was observed in all patients. Final measurements ranged 18.1–86.42 mm^2^. The increase in glottal area was statistically significant (*p* < 0.001), i.e., a mean of 23.16 mm^2^, obtaining about 327% of the original value.

A statistically significant postoperative increase in spirometric parameters was also observed, which was on average as follows: 1.18 L/s for PEF (*p* < 0.001), 0.53 L for FEV_1_ (*p* < 0.001), 0.51 L for FVC (*p*=0.001).

### 3.2. Self-Assessment of Respiratory Efficiency Using VAS and MRC

The self-assessment questionnaires on pre- and postoperative respiratory efficiency indicated a statistically significant reduction in VAS (*p* < 0.001) and MRC (*p* < 0.001) scores. Our analysis showed a significant postoperative improvement in breathing comfort (lower score) with increasing glottal area.

Voice quality analysis was not performed. After the surgery, some patients had a small amount of voice loss, however, all patients described their voices as socially efficient.

According to the medical records, immediately after the procedure, all patients complained of mild or moderate odynophagia. Some patients reported drinking difficulties on the 1st and 2nd day after surgery (choking), which, however, soon subsided, so that at the time of discharge from the department, they were able to consume solid and liquid foods on their own. There were no indications for rehabilitation in any of the patients.

### 3.3. CFD Simulation

**Figure 4:**
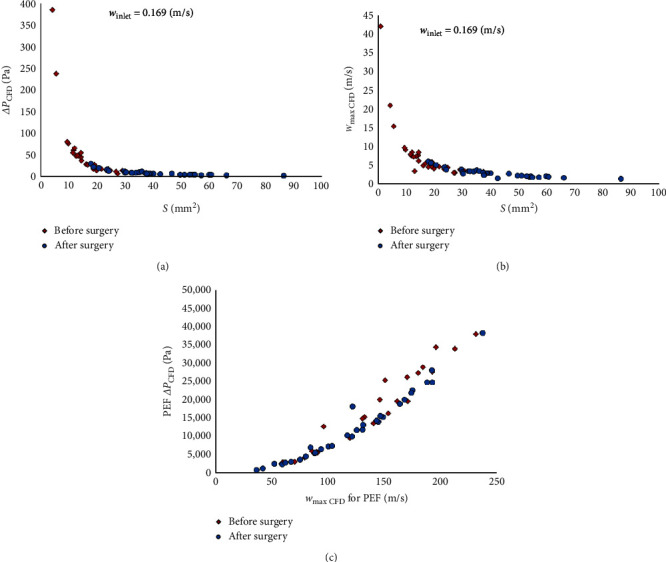
(a) *ΔP*_CFD_ pressure gradients for uniform inlet velocity *w*_inlet_ = 0.169 m/s for different glottic opening areas; (b) maximum air velocity *w*_max_ for different glottic opening sizes at *w*_inlet_ = 0.169 m/s; (c) comparison of pressure gradient PEF *ΔP*_CFD_ for different geometric shapes of glottic opening models and for PEF.

The measurements were performed for each patient, and the results are shown in Table 1 and Figure 4. One parameter, i.e., tidal volume (TV), was selected to compare glottal airflow at the same inlet velocity for all patients. This is a volume of air (0.5 L) that normally enters and leaves the lungs during quiet breathing. It was assumed that 10 breaths are taken in 1 min, which translated into an airflow of 5 L/min. This was converted from a fluid mechanics formula to obtain inlet velocity *w*_inlet_ = 0.169 m/s.

In our CFD analysis, we observed a postoperative reduction in maximum velocities at the glottal level (*w*_max CFD _*p* < 0.001, *w*_max CFD_ for PEF *p*=0.008).

The inlet velocity (*w*_inlet_ for PEF) values were significantly higher after the surgery (*p* < 0.001). This was accompanied by reduction in the intraglottal pressure gradients in both *ΔP*_CFD_ (*p* < 0.001) and PEF*ΔP*_CFD_ (*p*=0.002).

**Figure 5:**
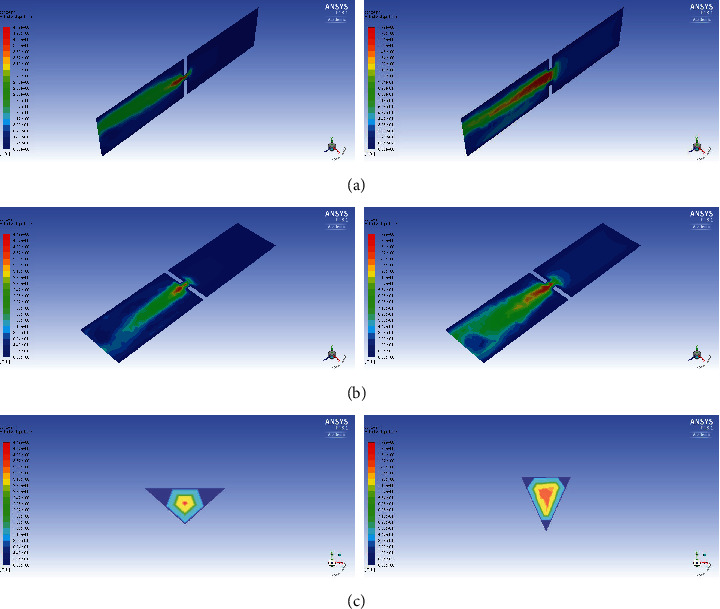
Velocity distribution fields for Patient No. 33 for preoperative PEF = 1.78 L/s (left) and postoperative PEF = 2.45 L/s (right): (a) in the *x* = 0 m plane; (b) in the *y* = 0 m plane; (c) in the *z* = 0.051 m plane (just below the glottis).

**Figure 6:**
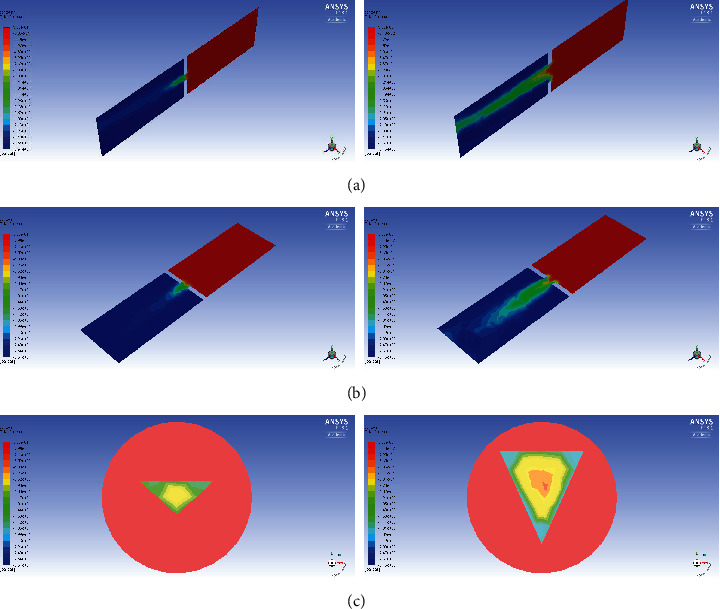
Pressure distribution fields for Patient No. 33 for PEF = 1.78 L/s before surgery (left) and for PEF = 2.45 L/s after surgery (right): (a) in the *x* = 0 m plane; (b) in the *y* = 0 m plane; (c) in the *z* = 0.051 m plane (just below the glottis).

The results of velocity and pressure gradients at the glottal level are presented in various planes using a computer-based model. Figures 5 and 6 show changes in the velocity and pressure gradients in Patient No. 33, who achieved a threefold increase in the glottal area after the procedure.

We found a significant negative correlation between *S* and PEF*ΔP*_CFD_, as well as between *S* and *ΔP*_CFD_. Therefore, higher glottal area values were accompanied by lower pressure gradients at the glottal level. Additionally, we found a significant positive correlation between PEF and PEF*ΔP*_CFD_, which means that higher PEF was accompanied by higher PEF-dependent pressure gradient.

An analysis of the relationships between the differences in the measurements showed that the change in the *S* parameter was significantly and negatively correlated with changes in VAS and MRC. It was also found that altered PEF*ΔP*_CFD_ was significantly positively correlated with altered FEV_1_ and PEF. This indicates that the higher the postoperative increase of glottal area, the higher the decrease of VAS and MRC scores; the higher the postoperative increase of PEF and FEV_1_, the lower the decrease of PEF-dependent pressure gradient.

## 4. Discussion

### 4.1. Surgical Effectiveness and Complications

To date, no single best technique for BVFP surgical treatment has been established. Endoscopic laser methods of unilateral arytenoidectomy, posterior cordectomy, and their combination are most widely approved by the medical community [2, 3, 11, 12]. These methods offer a comparatively high chance for improved respiratory efficiency and decannulation in some of the patients with tracheostomy. This paper may provide another evidence for good effectiveness of unilateral arytenoidectomy with posterior cordectomy in the treatment of BVFP. Satisfactory improvement of respiratory efficiency was achieved in 29 (87.87%) patients. Treatment failure was reported in four patients (12.12%).

The rate of revision surgeries is an important parameter for the assessment of surgical treatment. Complications requiring revision surgery reported by the authors range between 25% and 32% for patients after endoscopic laser expansion of the glottis [3, 13]. However, Bizakis et al. reported revision surgery rate of 0% after arytenoidectomy with posterior cordectomy performed on 18 patients with BVFP [14]. Nawka et al. assessed patients with BVFP who underwent posterior cordectomy, lateral fixation, and partial arytenoidectomy. Revision surgery rate during a 6-month follow-up (from primary surgery) was 25% and was associated with the need to eliminate granulation tissue from postoperative scar region [13]. To reduce the prevalence of early and late local complications, all of our patients received MMC application intraoperatively and postoperative steroid therapy. It is a common practice in many centers to routinely administer steroids after surgery [11, 14–16]. There are reports on the possible reduction of the risk of granulation tissue or adhesion formation in the wound by intraoperative local use of MMC. Damage of the laryngeal mucosa leads to fibroblast proliferation and collagen formation, which play a key role in scar formation. MMC shows anticancer and antiproliferative properties–it may inhibit fibroblast activity, fibrosis, and scarring. The use of MMC in laser expansion of the glottis and optimal MMC dosage is still under discussion [17–19]. In our study, complications requiring further surgical interventions were reported for 24.24% of patients. The presence of an adhesion was the major cause of secondary glottic stenosis. In this case, patients reported a characteristic course of convalescence, with significant respiratory improvement immediately after the surgery, which later decreased or completely resolved until follow-up examination (4−6 weeks after the procedure). Therefore, six patients underwent revision surgery, i.e., repeated laser dilatation of the glottis within 7–18 months of the primary surgery. One patient required a triplicate procedure. After the third revision surgery, tracheostomy was needed due to dyspnea; however, the patient was decannulated once the larynx wound healed. Ultimately, three patients in this group failed to achieve full therapeutic success that would allow for safe decannulation.

Two patients were qualified for Kleinsasser's directoscopy due to the presence of granulation tissue in the postoperative wound. The granulation tissue was removed within 3–5 months of the primary surgery with microtools, without the use of CO_2_ laser, achieving permanent outcomes.

Many authors consider decannulation to be an equivalent of therapeutic efficacy. It is often the actual goal of treatment for patients. Although some studies report a 100% rate of decannulation, this result should be interpreted with caution as analyses are often performed in small groups [14, 15, 20]. Bosley assessed a group of six patients with tracheostomy who underwent surgical widening of the glottic opening. Transverse cordotomy was performed in three patients, while the three other patients underwent medial arytenoidectomy, with decannulation performed in all patients. Bazakis included 18 patients in his analysis, three of whom had already had a tracheotomy, while the remaining patients had it performed simultaneously with arytenoidectomy with posterior cordectomy. None of the patients required revision surgery, and all of them were decannulated successfully within 2–3 weeks. Misiołek et al. published an analysis of the outcomes of laser arytenoidectomy with posterior cordectomy in 30 patients with a tracheostomy—all patients were decannulated. However, in his analysis of reports on the effects of various glottic dilation techniques (transient laterofixation, posterior cordotomy, partial arytenoidectomy, and combinations of these techniques), Nawka et al. concluded that decannulation is possible only in about 35% of patients with BVFP [3]. In our group of patients, tracheotomy was needed in nine patients. Decannulation was performed in 5/9 patients, whereas tracheostomy was maintained in 4/9 patients due to persisting dyspnea. We performed no analysis of risk factors for treatment failure, but it is highly probable that high body mass index (BMI) and a concomitant neurological disease limiting mobility (in one patient) contributed to treatment failure in our patients.

### 4.2. VAS and MRC Scores

There are many publications whose authors report marked improvement in respiratory efficiency after surgical glottic expansion. In most studies, the authors use no scores for the assessment of dyspnea reduction, but rather describe the “improvement” reported by their patients. Only a few studies used subjective Short Form Health Survey (SF-36) or MRC scores [8, 13, 21]. In our study, we used VAS and MRC scales, which are easy for patients to complete. The self-assessment questionnaires on pre- and postoperative respiratory efficiency indicated a statistically significant improvement expressed by 54% and 53% reduction in mean VAS and MRC scores. It was found that the change in the S parameter was significantly negatively and weakly correlated with changes in VAS and MRC parameters, which means that the greater the postoperative increase in the glottal area, the higher the decrease in the severity of dyspnea reported by patients.

A lack of improvement was observed in patients with preexisting tracheostomy, who could not be decannulated after glottal expansion. The increase in glottal area in these patients was insufficient for achieving satisfactory respiratory efficiency. Therefore, after tracheostomy tube removal and closure of the tracheostomy site, they still developed severe dyspnea at rest, as evidenced by the maximum VAS and MRC score. As mentioned before, it is highly probable that high BMI and a concomitant neurological disease limiting mobility (in one patient) contributed to treatment failure in those patients.

### 4.3. Spirometry

Spirometry is widely used by researchers for objective postoperative evaluation of BVFP patients [8, 9, 16, 22, 23]. In these patients, the narrow glottic opening causes upper airway dysfunction, and thus affects URT function parameters. Significant improvement of PEF, FEV_1_, PIF, and FEV_1_/FVC was observed after different glottis-dilating procedures (transverse cordotomy, partial or total arytenoidectomy, lateral fixation, and their combinations) [16, 22, 23]. Our analysis also revealed statistically significant postoperative increase in all spirometric parameters measured.

FEV_1_ is measured based on changes in the maximum flow over the entire lung volume range, and is therefore much less dependent on exertion and upper airway resistance compared to PEF. For this reason, in the case of upper airway obstruction, the reduction in PEF will be more pronounced than the reduction in FEV_1_. It has been suggested by some authors that the empey index (EI) and expiratory disproportion index (EDI), may be useful in the assessment of upper airway obstruction [24, 25]. In our study, EI and EDI upper airway obstruction criteria (EI > 10, EDI > 59) were met before and after surgical management by 60.6% and 51.5% of the 33 patients, respectively. Since glottis-dilating surgery is performed unilaterally in BVFP, the glottic opening does not reach the size seen in a healthy person. Therefore, the obstruction criterion is still met in terms of spirometric parameters despite significant respiratory improvement reported by patients [8]. However, it should be remembered that reduced dyspnea with the lowest possible risk of complications is the primary goal of treatment, and that bilateral surgical intervention significantly increases the risk of aspiration and deterioration of voice quality.

### 4.4. CFD Findings

#### 4.4.1. Glottic Model and Area

The glottic opening has a triangular shape in a healthy person with maximally abducted vocal cords. The mean glottal area is 100–150 mm^2^ in women and 190–250 mm^2^ in men [1]. Gökcan et al., in their CFD analysis, measured glottal area in 15 BVFP patients after bilateral laser transverse posterior cordectomy, obtaining values in the range of 29–90.2 mm^2^. As a result of the surgery, the glottal area increased to about half the surface area in controls. Neither subjective assessment of symptoms nor spirometry findings correlated with glottal area in this series [8]. In contrast, our analysis showed that the change in glottal area was significantly negatively correlated with changes in VAS and MRC parameters. As in our analysis, Gökcan also showed that although upper airway obstruction reduces postoperatively, it is still present, as confirmed by spirometry. According to the authors, patients most likely adapt to the new conditions by developing a more effective breathing technique. Gökcan found that the contours of the glottic opening are very important when determining the hydraulic diameter of the airways, and therefore the nature of the airflow (laminar or turbulent). However, the authors did not elaborate on this issue in their analysis. Marków et al. compared the shape of the glottis with isosceles triangles, equilateral triangles, and a semiellipse. The values of glottal area ranged between 18 and 64 mm^2^ in 24 BVFP patients after unilateral laser arytenoidectomy with posterior cordectomy and between 48 and 143 mm^2^ in five healthy controls. There has been a significant improvement in respiratory efficiency in patients with preoperative glottic opening smaller than 40 mm^2^, demonstrating a lower therapeutic value of glottis-dilating surgery in patients with glottic areas greater than 40 mm^2^ [9]. In 2019, Rios et al. assessed potential effects of virtual posterior cordotomy, lateral fixation, and posterior cricoid expansion (PCE) using larynx CFD models of three BVFP patients. The analysis in pre- and postoperative models showed that the maximum inspiratory velocity decreased with increasing glottal area. This correlation was also observed in our study. In Rios study, PCE allowed for a greater increase in the glottal area, as well as reduced airflow velocity and resistance; and the postoperatively achieved triangular shape (isosceles triangle) of the glottis may contribute to a more homogeneous distribution of airflow in the anterior-posterior axis, as pointed out by the authors. Further improvement of airflow resistance after additional glottic expansion was also observed in a BVFP patient with baseline glottal area of 50.4 mm^2^ [5]. In our study, we took into account both pre- and postoperative measurements. All patients qualified for the surgery met the *S* < 40 mm^2^ glottal area criterion. All patients underwent the same unilateral surgical procedure. Therefore, the final shape of the glottis largely depended on the position of the intact vocal fold. Due to the paramedian position of the vocal folds, which is most common in BVFP, the shape of an isosceles triangle dominated preoperatively. Because of the unilateral surgical intervention postoperatively dominated right angle triangle glottis shape.

Due to the retrospective nature of the study, we could only take into account the results of standard tests performed in our department in patients with BVFP. We intended to evaluate the pre- and postoperative results, therefore we analyzed the glottis fields on the basis of videolaryngoscopy, for which we adopted the abovementioned simplifications. CT could possibly bring more precise results, however, it is not performed on routine basis in BVFP patients due to lower availability and invasiveness. In our opinion, a separate comparative analysis of airflow through the glottis field, calculated on the basis of endoscopic images and the CT result, could be performed.

#### 4.4.2. Pressure and Velocity Measurements

Our surgical treatment led to a significant reduction in maximum velocities. We also observed postoperative drop in pressure gradients (Table 1). Pressure drop is an important parameter for airflow assessment as it has the potential to increase the risk of collapsing in the minimum cross-sectional area, especially when the wall is unstable, as in the case of BVFP. This was reflected in CFD analyzes in the form of significant drops in supraglottic pressure during peak inspiratory flow, which may cause tissue collapse at this site. Additionally, accelerated airflow generates turbulence and vortex structures, which reduce airflow effectiveness and increase respiratory effort [8]. This is also a limitation of our study as all models assumed rigid airway walls. Rios et al. [5] suggested a nonlinear relationship between the cross-sectional area and airflow velocity and resistance, as well as that there probably are “alteration points,” at which airflow parameters favorably increase.

## 5. Conclusions

In conclusion, unilateral laser arytenoidectomy with posterior cordectomy is an effective therapeutic modality for BVFP, allowing for a marked improvement in the respiratory efficiency of patients, which is expressed by increased spirometric parameters (PEF, FEV_1_, and FVC) and reduced VAS and MRC scores. The procedure allows for significant increase in the glottal area in BVFP patients. The CFD method is very useful for noninvasive visualization of laryngeal airflow in BVFP patients both before and after glottis-dilating surgery. It allows for a reliable assessment of surgical outcomes. CFD may show the accompanying changes in laryngeal airflow dynamics–reduced maximum velocity and pressure gradients. Moreover, we believe that CFD may be used to design the extent of the procedure necessary to obtain the optimal results, and the simple method of calculating the glottis field allows also for subsequent distant glottal airflow analysis based on endoscopic images and current spirometry.

## Figures and Tables

**Table 1:** Statistical results for measured parameters before and after the surgery.

Parameter	Operation dependency	*M*	*SD*	*Me*	*IQR*	Min	Max	*Sk.*
*S*	Before	18.67	8.49	18.04	11.27	1.2	37.73	0.14
	After	41.83	15.1	38.09	21.44	18.1	86.42	0.73

FEV_1_	Before	1.54	0.67	1.44	1.1	0.34	2.72	0.18
	After	2.07	0.66	2.02	0.91	0.55	3.44	−0.24

FVC	Before	2.38	0.97	2.56	1.47	0.4	4.09	−0.29
	After	2.89	0.85	2.98	1.08	1.26	4.88	0.17

PEF	Before	2.47	1.15	2.08	1.57	0.88	5.55	0.93
	After	3.66	1.26	3.63	1.33	0.62	6.33	−0.37

*w*_inlet for PEF_	Before	5.04	2.35	4.24	3.19	1.8	11.31	0.94
	After	7.45	2.57	7.4	2.72	1.26	12.9	−0.37

*w*_max for PEF_	Before	185.21	107.47	162.07	134.96	50.88	460.69	1
	After	126.89	62.01	122.65	78.86	37.36	341.21	1.22

PEF*ΔP*_CFD_	Before	46,747	81,723.3	20,109	28,906	2,195	446,989	3.85
	After	14,493	15,692.8	11,713	13,392	938	86,764	3.11

*w*_max CFD_	Before	7.39	7.24	4.97	3.49	2.93	42.09	3.7
	After	2.94	1.14	2.88	1.36	1.38	6.06	1.05

*ΔP*_CFD_	Before	170.77	711.13	26.47	40.52	6.23	4111	5.39
	After	7.54	6	6.1	5.67	1.36	28.88	1.91

VAS	Before	8.42	1.52	9	2	4	10	−1.02
	After	3.85	2.48	4	2	1	10	1.37

MRC	Before	4.3	0.77	4	1	2	5	−0.98
	After	2	1.2	2	1	1	5	1.44

## Data Availability

The data used to support the findings of this study are available from the corresponding author upon request.

## Abbreviations

BVFP: Bilateral vocal fold paralysis
CFD: Computational fluid dynamics
*ΔP*: Pressure gradient at the glottal level
*ΔP*_CFD_: Pressure gradient at the glottal level for uniform inlet velocity (*w*_inlet_ = 0.169 m/s)
URT: Upper respiratory tract
MRC: Medical Research Council
PCE: Posterior cricoid expansion
PEFR: Peak expiratory flow rate
PEF*ΔP*_CFD_: Pressure gradient at the glottal level for patients individual PEF value
RLN: Recurrent laryngeal nerves
*S*: Glottal surface area
VAS: Visual analogue scale
*W*: Airflow velocity
*w*_inlet_: Inlet air velocity at the glottal level
*w*_max_: Maximum velocity
*w*_max CFD_: Maximum velocity at the glottal level calculated using CFD for a predetermined inlet velocity of 0.169 m/s
*w*_max CFD for PEF_: Maximum velocity at the glottal level calculated using CFD for patient's individual PEF.
